# Discovery of Potent Disheveled/Dvl Inhibitors Using Virtual Screening Optimized With NMR-Based Docking Performance Index

**DOI:** 10.3389/fphar.2018.00983

**Published:** 2018-09-05

**Authors:** Kiminori Hori, Kasumi Ajioka, Natsuko Goda, Asako Shindo, Maki Takagishi, Takeshi Tenno, Hidekazu Hiroaki

**Affiliations:** ^1^Laboratory of Structural Molecular Pharmacology, Graduate School of Pharmaceutical Sciences, Nagoya University, Nagoya, Japan; ^2^Department of Biological Science, School of Science, Nagoya University, Nagoya, Japan; ^3^Division of Biological Science, Graduate School of Science, Nagoya University, Nagoya, Japan; ^4^Department of Pathology, Graduate School of Medicine, Nagoya University, Nagoya, Japan; ^5^BeCellBar LLC, Business Incubation Center, Nagoya University, Nagoya, Japan

**Keywords:** Wnt signaling, protein–protein interaction inhibitor, NMR-derived docking performance index, virtual screening, triple negative breast cancer

## Abstract

Most solid tumors have their own cancer stem cells (CSCs), which are resistant to standard chemo-therapies. Recent reports have described that Wnt pathway plays a key role in self-renewal and tumorigenesis of CSCs. Regarding the Wnt/β-catenin pathway, Dvl (mammalian Disheveled) is an attractive target of drug discovery. After analyzing the PDZ domain of human Dvl1 (Dvl1-PDZ) using NMR, we subjected it to preliminary NMR titration studies with 17 potential PDZ-binding molecules including CalBioChem-322338, a commercially available Dvl PDZ domain inhibitor. Next, we performed virtual screening (VS) using the program GOLD with nine parameter sets. Results were evaluated using the NMR-derived docking performance index (NMR-DPI). One parameter set of GOLD docking showing the best NMR-DPI was selected and used for the second VS against 5,135 compounds. The second docking trial identified more than 1,700 compounds that exhibited higher scores than CalBioChem-322338. Subsequent NMR titration experiments with five new candidate molecules (NPL-4001, 4004, 4011, 4012, and 4013), Dvl1-PDZ revealed larger chemical shift changes than those of CalBioChem-322338. Finally, these compounds showed partial proliferation inhibition activity against BT-20, a triple negative breast cancer (TNBC) cell. These compounds are promising Wnt pathway inhibitors that are potentially useful for anti-TNBC therapy.

## Introduction

Poor therapeutic outcomes of chemotherapy against several solid tumors pose a challenge to anti-tumor drug discovery and development. Cancer stem cells (CSCs) are believed to have a pivotal role in malignancy, survival against chemotherapy, and self-renewal of those tumors (Tannishtha et al., 2001). Consequently, CSCs are attractive targets for cancer chemotherapy development (Visvader and Lindeman, 2008). The Wnt/β-catenin pathway, along with Notch and Hedgehog pathways, are important in several CSCs (Reya and Clevers, 2005). In fact, the Wnt pathway has been commonly regarded as the key signaling pathway of self-renewal and anti-differentiation of normal tissue stem cells. Accordingly, proliferation and self-renewal of several CSCs have been demonstrated as dependent on the Wnt pathway. For that reason, Wnt pathway is an attractive target for anti-CSC chemotherapy (Takebe et al., 2011; Holland et al., 2013).

The Wnt/β-catenin pathway is activated by Wnt ligands. Frizzled (Fzd)/LRP co-receptors coordinately bind Wnt, and transduce the signal to cytosolic downstream components including Axin, APC, GSK3β, and CK1. Accordingly, a transcription factor β-catenin is accumulated to induce target gene expression. This signaling system is carried out by a constitutive process of proteasomal degradation of β-catenin at the “Wnt-off” state. Specifically, β-catenin degradation is initiated by phosphorylation by GSK3β. At “Wnt-on” state, then the interaction between Dvl, and Axin inhibits GSK3β, thereby accumulating β-catenin in the cytoplasm and the nucleus. Dvl, a 75 kD multi-domain adaptor protein with Disheveled-aXin (DIX), Post synaptic density-95, Disc large, and Zonular occludens-1 (PDZ), and Disheveled-Egl10-Pleckstrin (DEP) domains (**Figure 2A**), plays a central role in both canonical (β-catenin-dependent) and non-canonical (β-catenin-independent) pathways of Wnt signaling (Gao and Chen, 2010). There are three mammalian Disheveled orthologs, Dvl-1, 2, and 3, in human genome, with functional redundancy. The PDZ domain of Dvl (Dvl-PDZ) specifically interacts to the C-terminus of Fzd (Wong et al., 2003) upon Wnt binding to the extracellular domain of Fzd. Accordingly, Dvl-PDZ is an attractive target for exploring small molecule inhibitors (**Figure 2B**), and has been characterized extensively. For instance, the binding mode of the tripeptides VVV and VWV against Dvl-PDZ has been reported (Lee et al., 2009a). The complex structure of peptide-derived inhibitors and Dvl2-PDZ has also been reported (Zhang et al., 2009). In addition, several reports have described Dvl-PDZ inhibitors, including a peptide-mimic compounds NSC668036 (Shan et al., 2005), 1H-indole-5-carboxylic acid derivative FJ9 (Fujii et al., 2007), sulindac (Lee et al., 2009b), *N*-benzoyl-2-amino-benzoic acid derivative CalBioChem-322338 (Grandy et al., 2009), and phenoxyacetic acid analogs (Choi et al., 2016). The present study specifically examines *N*-benzoyl-2-amino-benzoic acid analogs including CalBioChem-322338 because 2-amino-benzoic acid moiety is independently proposed as a key moiety of group-specific inhibitors against several PDZ domains. Therefore, it represents a potential pharmacophore (Tenno et al., 2013). During our research exploring new inhibitors against Zonular Occludens-1 PDZ1 domain (Umetsu et al., 2011), we obtained several *N*-substituted-2-amino-benzoic acid analogs that are chemically similar to CalBioChem-322338 (**Figure 3** and **Supplementary Figure S1**). The present study evaluates the affinities of those compounds against human Dvl1 PDZ domain (hDvl1-PDZ) using solution NMR experiments (**Figure 2D**).

Virtual screening (VS) of drug candidates, known as high-throughput protein–ligand docking, is a powerful approach. Commercial applications are widely used, such as Glide (Friesner et al., 2004), FRED (McGann, 2011), MOE/ASEDock (Goto et al., 2008), and GOLD (Verdonk et al., 2003), as well as academic applications such as AutoDock (Goodsell et al., 1996), AutoDock-VINA (Trott and Olson, 2010), and Sievegene (Fukunishi et al., 2005). According their increasing convenience and availability, another practical issue has arisen: VS experiments with different algorithms, different parameter settings, and different target 3D structures might produce disparate results. Consequently, the benchmarking of docking algorithms has come to represent an important issue (McGaughey et al., 2007; Lindh et al., 2015). For the present study, we decided to use GOLD because GOLD is recognized as having acceptably high performance in comprehensive benchmarking throughout several VS programs (Wang et al., 2016). Moreover, results have demonstrated that the experimental tuning of parameter sets and/or the selection of target model structures might greatly improve performance and provide higher accuracy of prediction (Huang and Wong, 2016). Encouraged by that idea proposed by Huang et al., we introduced the idea into our project as a simplified index for evaluating nine docking scoring functions of GOLD. For this study, the index is designated as the NMR-based docking performance index (NMR-DPI).

**FIGURE 1:**
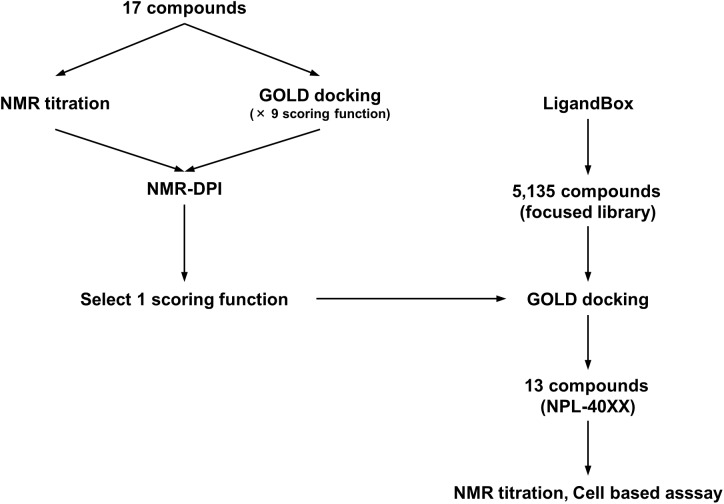
Schematic overview of optimization of the GOLD scoring function by using NMR-DPI and the subsequent virtual screening of Dvl inhibitors.

First, 17 potential PDZ-binding molecules as well as CalBioChem-322338, all of which are *N*-substituted-2-amino-benzoic acid analogs, were analyzed using NMR chemical shift perturbation (CSP) experiments. We believe that the NMR-CSP experiment is among the easiest and most robust assay methods to compare the affinities of a series of compounds against ^15^*N*-labeled small protein (Williamson, 2013). Second, these 17 potential PDZ-binding molecules were docked against hDvl2-PDZ using GOLD with nine different scoring functions. Third, out of the nine scoring functions, we identified the one that is most consistent to the CSP experiments of the 17 compounds. This optimized scoring function was used for a new VS with our in-house focused library, which is a subset of the library LIGANDBOX (Kawabata et al., 2013) containing commercially available 5,135 *N*-substituted-2-amino-benzoic acid analogs. From the top hit compounds after the new VS experiment, 13 new molecules were purchased: NPL-4001 – NPL-4007, and NPL-4011 – NPL-4016 (**Figure 1**). Our seven original compounds induced markedly larger chemical shift changes upon hDvl1-PDZ than those induced by CalBioChem-322338. The compounds were evaluated further by the cell-based assays as potential Wnt pathway inhibitors. The validity and possible limitations of NMR-DPI were also assessed.

## Materials and Methods

### Preparation of Protein Samples

The expression vector for the recombinant GST-tagged form of prototype hDvl1-PDZ^∗^ domain (residues 244–342) was constructed using the PRESAT-vector methodology (Goda et al., 2004). The vector for the GST-tagged hDvl1-PDZ domain (residues 246–340, four amino acids shorter construct) was then produced using the standard PCR cloning technique with pGEX-6P3 plasmid (GE Healthcare, Little Chalfont, United Kingdom). The GST-tagged hDvl2-PDZ domain (residues 262–356) was constructed similarly. Two PDZ domains, residues Cys-Trp near the C-termini (residues 338–339 and 354–355, respectively, for hDvl1, and hDvl2) were substituted to Ala-Thr to increase protein stability. Since the position of these residues was opposite side to the ligand binding site, we assumed that the mutations affected to neither its affinity nor binding mode to the inhibitors. Isotopically labeled proteins for NMR experiments were generated, respectively, in *E. coli* BL21 (*DE3*) grown in 1 L M9 minimal medium culture at 37°C in the presence of [^15^N]-NH_4_Cl and [^13^C]-glucose (if needed) as the sole nitrogen and carbon sources. The protein expression was induced by addition of final 1 mM of isopropyl-β-D-galactoside, with immediate lowering of the temperature to 20°C. The cells were harvested 20 h after IPTG induction. The harvested cells were then re-suspended in lysis buffer (50 mM Tris–HCl, pH 7.2, and 150 mM NaCl), disrupted by sonication, and clarified by centrifugation. The supernatant was applied to a DEAE–Sepharose^TM^ Fast Flow (GE Healthcare) column. It was then affinity-purified using resin (GST-Accept^TM^; Nacalai Tesque Inc., Kyoto, Japan). The GST tag was removed by PreScission protease on beads. The protein solution was loaded on a Superdex 75 HR 26/60 column (GE Healthcare) equilibrated with 50 mM Tris–HCl (pH 7.2) and 150 mM NaCl. The purified proteins were concentrated to 0.1 mM (for NMR titration experiment) and were dialyzed against 100 mM potassium phosphate buffer (pH 7.4) containing 0.5 mM EDTA supplemented with 10% D_2_O and 5% d_6_-dimethyl sulfoxide. After comparing ^1^H-^15^N HSQC spectra of hDvl1 and hDvl2 PDZ domains, we decided to continue further study of hDvl1-PDZ because of its sharp and well-dispersed HSQC signals. For triple resonance experiments, 0.65 mM of ^15^N /^13^C-labeled hDvl1-PDZ was solubilized into 90 mM potassium phosphate buffer (pH 7.4) containing 0.45 mM EDTA supplemented with 10% D_2_O. ^15^*N*-labeled mouse ZO-1 first PDZ domain (residues 18–110, mZO1-PDZ1) was prepared according to an earlier report (Umetsu et al., 2011).

### NMR Experiments

For this study, NMR experiments were conducted using NMR spectrometer (600 MHz, Bruker Avance III; Bruker Analytik GmbH, Karlsruhe, Germany) equipped with a cryogenic triple-resonance probe. For assignment of backbone ^1^H, ^13^C, and ^15^N resonances, HNCA, HNCACB, CBCA (CO) NH, HNCO, HN (CA) CO, and 3D ^15^N-edited-NOESY-HSQC spectra were recorded. For NMR titration experiments, 0.1 mM PDZ domain sample was dissolved in 250 μL of 85 mM potassium phosphate buffer (pH 7.4) containing 0.42 mM EDTA supplemented with 10% D_2_O and 5% d_6_-dimethyl sulfoxide (DMSO). Then the ^1^H–^15^N HSQC spectra were obtained with and without ligands. In each titration experiment, the final concentration of the compound at 0.2 mM was added to the proteins. All NMR spectra were recorded at 298 K. All spectra were processed using NMRPipe (Delaglio et al., 1995) and were analyzed using the program Sparky 3.114 (Goddard and Kneller, 2004). All chemical shift changes in the ^1^H–^15^N HSQC spectra were calculated as Δδ_*normalized*_ = {Δδ(^1^H)^2^ + [Δδ(^15^N)/6]^2^}^1/2^. The chemical shift changes were then mapped onto the corresponding residues of the structure of hDvl2-PDZ using PyMol graphic software (Schrödinger, 2015). Δδ_*ave*_ is the sum of Δδ_*normalized*_ divided by the total residue number with their residue-specific assignment except the residues with broadened-out signals. After Signals showing marked chemical shift changes were selected, the normalized chemical shift changes were calculated. Non-linear least-squares fitting was applied to estimate the dissociation constant *K_D_* as

(1) $$\begin{array}{lll} \Delta \delta_{\mathrm{normalized}} & = & \Delta \delta_{\mathrm{saturated}}\times (({\left[R\right]}_{\mathrm{total}}+{\left[L\right]}_{\mathrm{total}}+K_{D})-\\ & & \mathrm{sqrt}({\left({\left[R\right]}_{\mathrm{total}}+{\left[L\right]}_{\mathrm{total}}+K_{D}\right)}^{2}-4{\left[R\right]}_{\mathrm{total}}\\ & & {\left[L\right]}_{\mathrm{total}}))/2{\left[L\right]}_{\mathrm{total}} \end{array}$$

where Δδ*_saturated_* represents the normalized chemical shifts at the saturated point. In addition, [*R*]*_total_* and [*L*]*_total_*, respectively, denote the concentrations of PDZ domain and the compound. *K_D_* and Δδ*_saturated_* values for the selected residues were optimized simultaneously by using SOLVER function in Microsoft Excel (Microsoft Corp.).

### Docking and Virtual Screening Experiments

Prior to the VS experiments, a focused library was constructed by filtering compounds with carboxylic acid moieties, which play a crucially important role in canonical peptide recognition by many PDZ domains. A focused library was constructed as a subset of the compound database (LIGANDBOX ver. 1306) (Kawabata et al., 2013) based on our earlier observation that diclofenac and flufenamic acid bound several PDZ domains in a group-specific manner (Tenno et al., 2013). We selected and pooled 5,135 compounds of *N*-substituted 2-amino-benzoic acid and *N*-substituted 2-amino-benzeneacetic acid. Subsequently, software GOLD suite (ver. 5.32) (Verdonk et al., 2003) was used for molecular docking of the compounds into the structure of hDvl2-PDZ [PDB entry 3CBY (Zhang et al., 2009)]. The GOLD software is based on a genetic algorithm for generating configurations of ligands with the two scoring modes, “simple scoring” and “consensus scoring.” Simple scoring uses just a single function out of the four fitness functions. Consensus scoring combines two of four scoring functions, respectively, for initial docking and re-scoring. The present study examined the three scoring functions of ChemScore (CS), GoldScore (GS), and ChemPLP, in the simple scoring mode and the consensus scoring mode, thereby examining nine scoring methods.

### Cell-Based Viability Assay

The newly found Dvl-PDZ inhibitors were tested to assess their effectiveness against TNBC cell lines (BT-20) on cell proliferation and viability. For that purpose, luciferase-expressing stable cell lines were chosen, although we did not perform luciferase-based biochemical experiment in this report. The TNBC cell lines BT-20 (BT-20/CMV-Luc, JCRB-1438) were obtained from the JCRB Cell Bank, National Institute of Biomedical Innovation, Health, and Nutrition (Osaka, Japan). The cells were grown in Minimum Essential Medium Eagle (Earle’s salts containing with **L**-glutamine and sodium bicarbonate; Sigma-Aldrich Corp.), supplemented with 10% fetal bovine serum (FBS) (Biosera, Boussens, France), and 1% Penicillin/Streptomycin antibiotics (Gibco, Grand Island, NY, United States). Cell lines were cultured in a 37°C incubator with a humidified atmosphere of 5% CO_2_. Cells were seeded at 15,000 cells/well into 96-well plates. After overnight incubation, cells were treated with d_6_-DMSO or 100 μM of each Dvl-PDZ inhibitor (CalBioChem-322338, NPL-4001, 4002, 4007, and 4011–4013) for 96 h. During culture, the media with or without corresponding inhibitors was refreshed every 48 h. After 4 days of culture with the compounds, the cell growth rate was ascertained using WST-8 [2-(2-methoxy-4-nitrophenyl)-3-(4-nitrophenyl)-5-(2,4-disulfophenyl)-2H-tetrazolium] colorimetric assay with a kit (Cell Counting Kit-8^®^; Dojindo Molecular Technologies Inc., Kumamoto, Japan) according to the manufacturer’s instructions. Cell viability was also ascertained after 4 days (Cytotoxicity LDH Assay Kit-WST; Dojindo Molecular Technologies Inc., Japan). The sample absorbance was measured using a microplate reader (EnSpire; PerkinElmer Inc., Waltham, MA, United States). All experiments were performed in triplicate. Each measurement was repeated twice. Statistical tests were performed using Microsoft Office^®^ Excel program.

## Results

### NMR Analysis of hDvl1-PDZ With Prototype *N*-Substituted 2-Amino-Benzoic Acid Compounds

**FIGURE 2:**
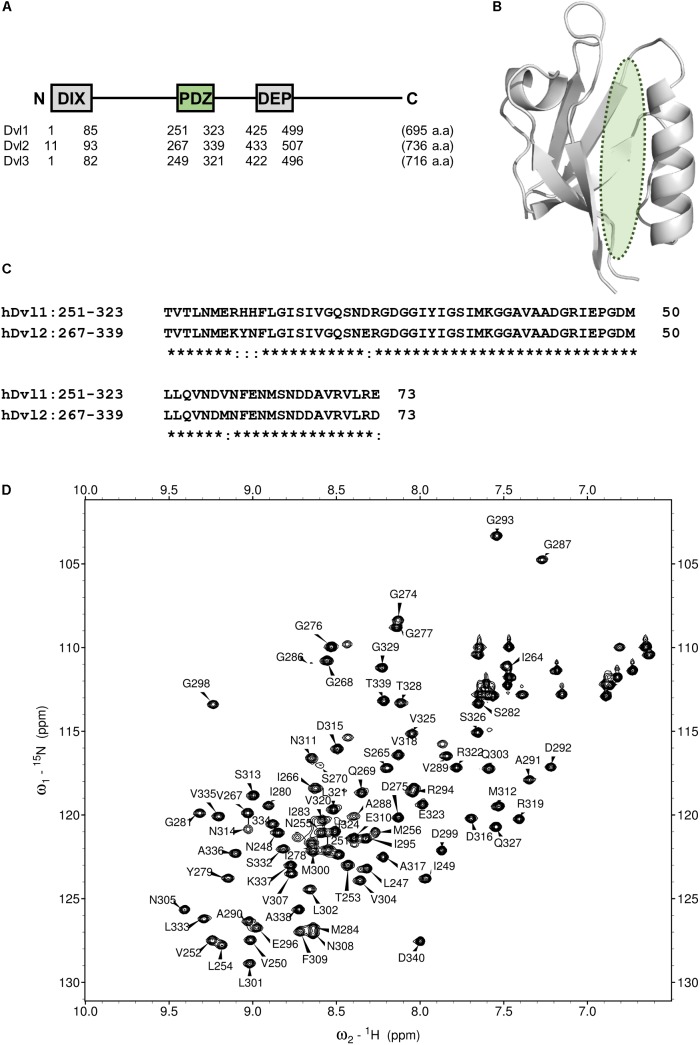
PDZ domain of Dvl as a drug target for Wnt pathway. **(A)** Domain architecture of three human Disheveled orthologs. DIX, DIsheveled-aXin domain, PDZ, Post synaptic density-95, Disc large and Zonular occludens-1domain, DEP, Disheveled-Egl10-Pleckstrin domain. **(B)** Ribbon representation of PDZ domain of Dvl2 (PDB code: 3CBY). A green ellipsoid indicates the position of Fzd binding cleft. **(C)** Multiple sequence alignment of the core region of PDZ domain of human Dvl1 (residues 251–323) and Dvl2 (residues 267–339). Identical amino acids are represented by asterisks. **(D)** A portion of the ^1^H–^15^N HSQC spectrum of hDvl1-PDZ illustrating a number of the assigned backbone amide resonances.

Before analyzing the interaction between hDvl1-PDZ and the compounds, we completed assignment of the backbone amide signals of hDvl1-PDZ because few signal assignments for hDvl1-PDZ have been published or deposited in the public NMR database (BioMagResBank). The backbone signal assignment was done according to the standard method (Ikura et al., 1990) using software MARS (Jung and Zweckstetter, 2004). The assignment was further confirmed using several inversely ^14^*N*-labeled samples (Hiroaki et al., 2011). Out of the 98 residues, 79 residues (81%) were assigned, although seven NH signals at the loop between β1 and β2 strands were missing, probably because of intermediate dynamic motion in the solution. The assignments were labeled on the HSQC spectra (**Figure 2D**).

**FIGURE 3:**
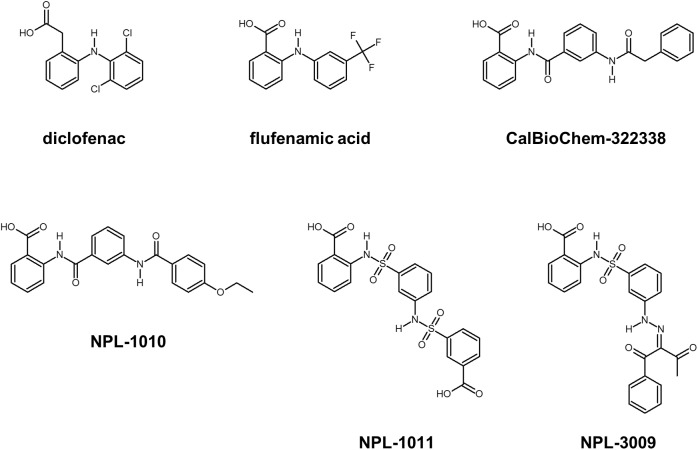
Selected compounds that may bind PDZ domains. Diclofenac and flufenamic acid bind several PDZ domains. CalBioChem-322338 is an example of Dvl-PDZ inhibitors. NPL-1010, 1011, and 3009 are an example of potential hDvl1-PDZ binding compounds.

**FIGURE 4:**
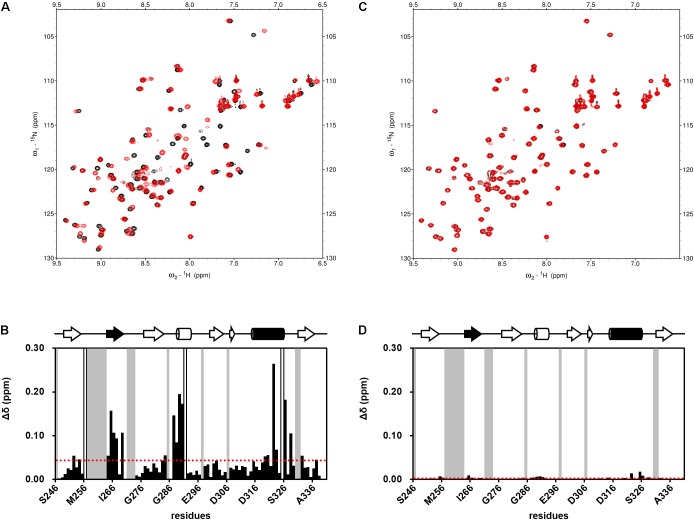
Examples of NMR titration of PDZ domains with compounds **(A,C)** and their normalized chemical shift changes **(B,D)**. **(A,B)** NPL-3009. **(C,D)** NPL-3003. **(A,C)** each overlaid spectrum was derived from 0.1 mM hDvl1-PDZ with (red) and without (black) the 0.2 mM of compound. The signals with markedly large CSPs were boxed and indicated with the residue numbers. **(B,D)** normalized chemical shift changes Δδ is plotted against residue numbers. Gray residues are missing or unassigned residues, and white residues indicate Pro. The secondary structure of hDvl1-PDZ is shown at the top of the figures, whereas α2 and β2 are shown in black.

Subsequently, we performed NMR titration experiments using 17 prototypical *N-*substituted 2-amino-benzoic acid compounds (NPL-1010, 1011, and 3001–3015) (**Figure 3**). In an earlier study, we found from bioinformatics prediction of the eF-seek analysis of all PDZ domains in human genome (Kinoshita et al., 2007; Motono et al., 2011), that flufenamic acid and diclofenac bound several PDZ domains (Tenno et al., 2013). Moreover, we identified the structure of the mouse Zonula ocludens-1 (ZO1)-PDZ1 domain (Umetsu et al., 2011) (PDB: 2RRM) and mouse ligand of numb X1 (LNX1)-PDZ2 domain (PDB: 3VQG, 3VGF, manuscript in preparation). These structures were subjected to VS using GOLD and LIGANDBOX to discover novel PDZ domain inhibitors. During that study, we identified the first two prototypical mLNX1-PDZ2 binders (NPL-1010 and 1011), for which direct binding to mLNX1-PDZ2 was confirmed using NMR experiments (manuscript in preparation). Surprisingly, the chemical structure of NPL-1010 closely resembled that of CalBioChem-322338 (**Figure 3**). Accordingly, we proceeded to collect 15 related compounds (NPL-3001–3015) to analyze affinities against both mLNX1-PDZ2 and mZO1-PDZ1 by the combined use of VS and solution NMR. Subsequently, our collected *N-*substituted 2-amino-benzoic acid compounds (NPL-1010, 1011, and 3001–3015) were examined to elucidate whether they bind directly to hDvl1-PDZ, or not. Finally, we found that 12 of 17 compounds tested in this study showed substantial chemical shift changes of amide protons of hDvl1-PDZ larger than that of CalBioChem-322338. All results of chemical shift changes were normalized and were averaged per residue. They are presented in **Table 1** according to descending order of the CSPs. Examples of the chemical shift perturbations are presented in **Figure 4**.

**Table 1:** Normalized total CSPs of hDvl1-PDZ induced by 2.0 equations of the prototypical Dvl1-PDZ binding compounds.

Compound ID (NPL-)	Δδ_*ave*_/ppm	Compound ID (NPL-)	Δδ_*ave*_/ppm
1010	0.022	3008	0.032
1011	0.022	3009	0.044
3001	0.021	3010	0.027
3002	0.007	3011	0.022
3003	0.003	3012	0.021
3004	0.012	3013	0.022
3005	0.023	3014	0.015
3006	0.019	3015	0.017
3007	0.022	CalBioChem-322338	0.018

### Introduction and Calculation of NMR-Derived Docking Performance Index

**FIGURE 5:**
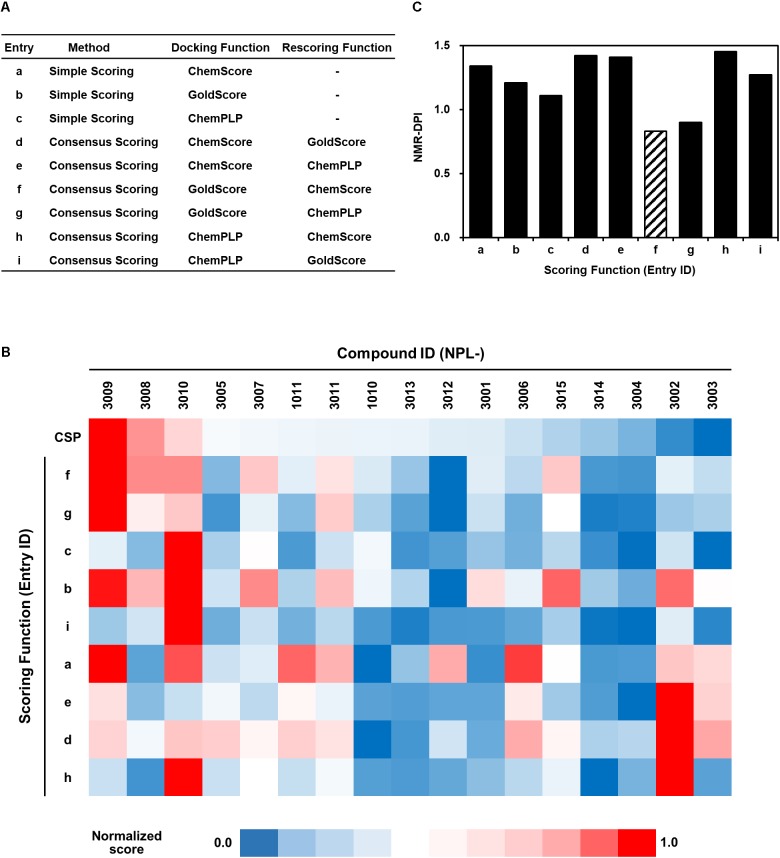
Full list of GOLD scoring functions used in this study **(A)**, a heat map representation of the corresponding GOLD docking scores of NPL-30XX compounds **(B)**, and their NMR-DPIs **(C)**. Similarity and difference are represented as a heat map. Each normalized score of NMR chemical shift perturbation (CSP) and GOLD scoring functions (a–f, same as in **(A)** are colored with red (score = 1.0) to navy (score = 0.0). The GOLD scoring functions are in the order of similarity.

Greatly inspired by the idea of fine-tuning of VS parameters and setting them with experimental data to improve VS performance (Huang and Wong, 2016), we modified that original idea to fit the use of our experimental data of NMR titration (CSP) study. For this purpose, we designed a strategy to tune VS parameters with our original NMR-derived docking performance index (NMR-DPI, **Figure 1**). First, NMR titration experiments of hDvl1-PDZ were performed with all 18 compounds as described above. Second, we docked all the 17 *N-*substituted 2-amino-benzoic acid compounds to the hDvl2-PDZ structure (PDB: 3CBY) using the GOLD software. Note that the core region of PDZ domains of human Dvl1 and Dvl2 are 92% identical in amino acid sequences (**Figure 2C**). At that time, the nine docking scoring methods were tested with different combinations of scoring functions, as presented in Table in **Figure 5A**. In our experience, these GOLD scoring functions mutually differ to a great degree. For that reason, it is difficult to determine one of them robustly for any new VS project. Third, the final fitness score of each scoring method was normalized to a value between 0 and 1 as the docking score *D*(*i, j*), where *i* is the index of the scoring methods and *j* is the name of the compounds. Similarly, the averaged normalized NMR chemical shift change, *N*(*j*), was calculated. Finally, NMR-DPI was defined as

(2) $$\text{NMR}\_\text{DPI}(\text{i})=\mathrm{sqrt}\left(\sum_{}^{j}{\left(D(i,j)-N(j)\right)}^{2}\right)$$

The heat map representation of all docking scores of the 17 compounds with nine scoring functions in GOLD and the normalized averaged NMR chemical shift change for 18 compounds is shown in **Figure 5B**. A bar graph of NMR_DPI is portrayed in **Figure 5C**. The lowest NMR_DPI, which represents the best correlation between the docking score and the NMR CSP experiments, was achieved when the consensus scoring of GS followed by CS was selected.

### Advanced Virtual Screening of hDvl1-PDZ Domain Inhibitors

**FIGURE 6:**
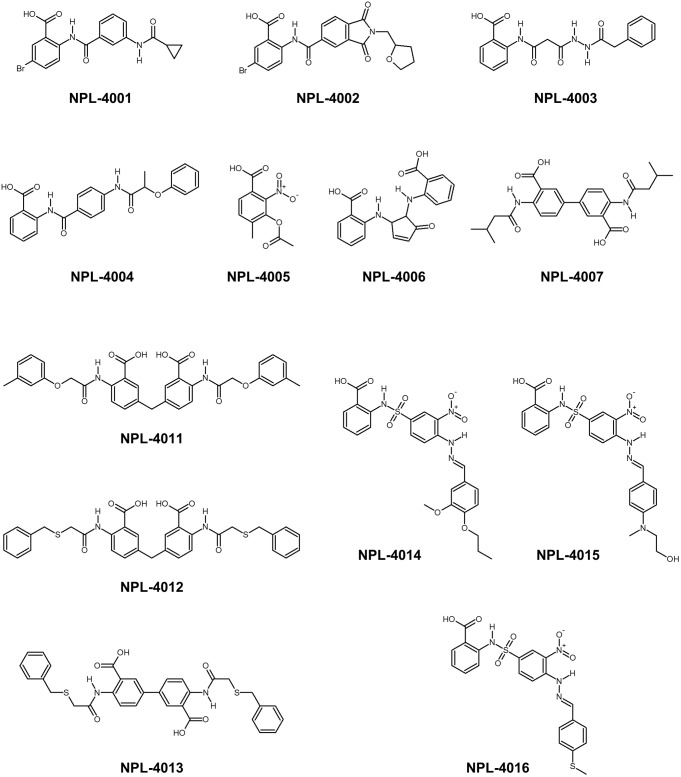
Chemical structure of newly found Dvl-PDZ inhibitor candidates.

Consensus scoring GS-CS in this order was chosen to perform the advanced VS experiment with GOLD and the specified library, including approximately 5,135 *N*-substituted-2-amino-benzoic acid compounds. We obtained a list containing 1,770 compounds with scores higher than that of CalBioChem-322338 (score = 59.9). After the selected compounds were purchased (**Figure 6**), they were assessed using NMR-CSP experiments to ascertain whether they were able to bind hDvl1-PDZ. Among them, nine compounds (NPL-4001, 4002, 4004, 4007, and 4011–4016) induced substantial chemical shift changes when added to hDvl1-PDZ: 7 out of 13 (69%) compounds had reasonable affinity against hDvl1-PDZ (**Supplementary Table S1**). Some HSQC spectra are presented in **Supplementary Figure S2** with their chemical structures. The hit rate (69%) is remarkably high, emphasizing the benefit of introducing NMR-DPI combined with VS.

### Assessing Physicochemical Properties of the Most Potent hDvl1-PDZ Inhibitor: NPL-4011

**FIGURE 7:**
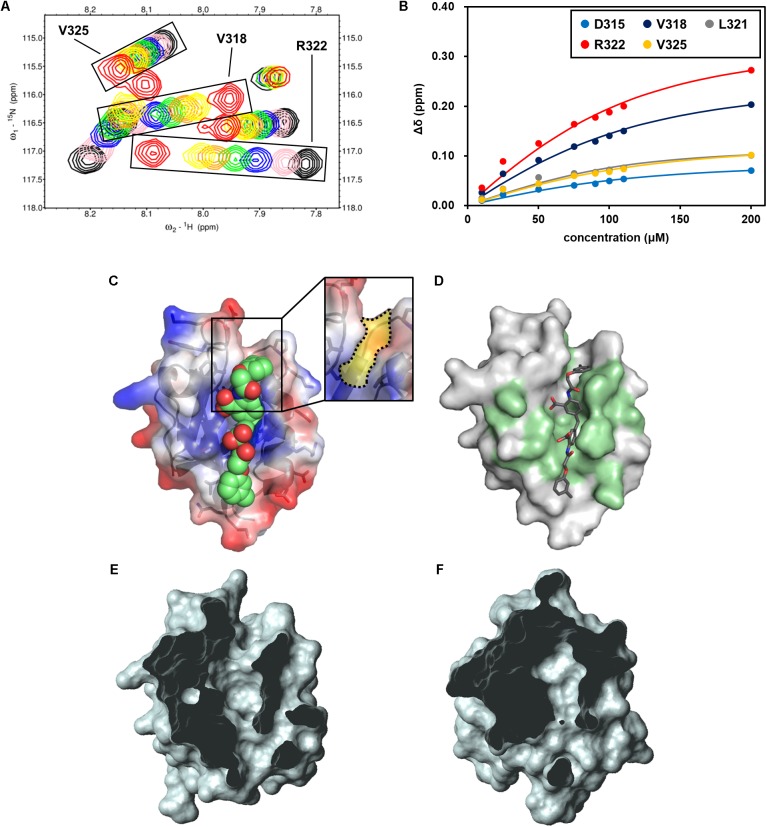
NMR titration experiment of hDvl1-PDZ with NPL-4011. **(A)** Expanded region of ^1^H–^15^N HSQC spectra of hDvl1-PDZ with 0 equation. (black), 0.25 equation. (pink), 0.5 equation. (navy), 0.75 equation. (green), 1.0 equation. (orange), 1.2 equation. (yellow), and 2.0 equation. (red) of NPL-4011 were overlaid. The assignments of the signal series are labeled. **(B)** Normalized chemical shift changes of the selected hDvl1-PDZ residues upon titration with NPL-4011. Solid lines indicate the non-linear fitting curves of each signals based on the single-site binding model. **(C)** Example of docking models of hDvl2-PDZ with NPL-4011 predicted by GOLD. The Dvl-PDZ unique cleft is boxed and colored yellow (inset). **(D)** CSPs induced by NPL-4011 binding are mapped on the surface structure of hDvl2-PDZ. **(E,F)** Surface representations of hDvl2-PDZ **(E)** and mouse ZO-1 PDZ1 **(F)** clipped by a front plane. The clipping size is 13.5 Å and the clipped position is 6.5 Å. Many PDZ domains do not possess the groove corresponding to Dvl-PDZ unique cleft which was clearly seen in **(E)**.

Among the 13 newly examined compounds, four (NPL-4007, 4011, 4012, and 4013) possessed a common molecular architecture, with two 2-amino-benzoic acid moieties connected at the 5-position directly or with a single methylene linker (**Figure 6**). NPL-4011 showed a large GOLD VS docking score as well as CSP. Therefore, we determined its *K_D_* further against hDvl1-PDZ using NMR titration experiments (**Figure 7A** and **Supplementary Figure S3A**). First we selected the residues surrounding the ligand binding pocket: D315, V318, L321, R322, and V325. The normalized chemical shift changes of these residues were subjected to non-linear curve fitting to find *K_D_* (**Figure 7B**), which was 34.5 ± 6.6 μM. Then we compared this value to the commercially available control compound CalBioChem-322338 under the same condition and obtained the value of 954 ± 403 μM (**Supplementary Figures S3C,D**). This *K_D_* value of CalBioChem-322338 is larger than its reported value for mouse Dvl1-PDZ (10.6 ± 1.7 μM) (Grandy et al., 2009) for reasons that remain unknown. Results show that NPL-4011 is a stronger inhibitor than CalBioChem-322338 when compared under identical conditions using hDvl1-PDZ.

Next, we carefully assessed the docking model of NPL-4011 and Dvl-PDZ generated by GOLD (**Figure 7C**). In the model, the crescent-shaped molecule NPL-4011 is well suited to the long shallow cleft of the ligand binding site of Dvl-PDZ. The residues of hDvl2-PDZ which contact to NPL-4011 are consistent with the residues that showed substantial CSPs at the NMR titration experiments (**Figure 7D**). We examined this binding model further. The lower half part of the symmetrical NPL-4011 molecule fits to the lower half part of the ligand binding cleft of Dvl-PDZ, which corresponds to the “canonical” C-terminal binding pocket common for all other PDZ domains. The upper half part of NPL-4011 also fits to the cleft between two loops: β1–β2 loop and α2-β6 loop. This upper cleft is unique to Dvl-PDZ domain (**Figures 7D,E**), which might accommodate binding to “non-canonical” ligands such as the cytosolic regions of Fzd, the physiological partner of Dvl. **Figure 7F** is an example of a close-up view of the representative “canonical” class-III PDZ domain, the first PDZ domain of mouse ZO-1 (mZO1-PDZ1, PDB:2RRM). The domain does not possess the cleft above the canonical ligand binding pocket because the loop between β1–β2 bends upon and contacts to the end of α2-helix.

This structural difference between Dvl-PDZ and mZO1-PDZ1 invites our speculation that, because of steric crash between the half part of the ligand and the bended β1–β2 loop, NPL-4011 (and probably its related molecules, NPL-4007, 4012, and 4013) might not bind mZO1-PDZ1. Instead, the smaller prototype Dvl-PDZ inhibitor CalBioChem-322338 can bind mZO1-PDZ1 because it might only occupy the canonical ligand binding pocket of mZO1-PDZ1 without steric stress. In other words, NPL-4011 is among the more Dvl-specific PDZ domain inhibitors. In order to confirm this speculation, we further performed additional NMR-CSP experiments of mZO1-PDZ1 titrated with NPL-4011 and CalBioChem-322338 (**Supplementary Figure S4**). Assignment of backbone signals were taken from our previous study (Umetsu et al., 2007). In the presence of two equivalent of NPL-4011, mZO1-PDZ1 did not show any chemical shift changes. In contrast, the signals from the residues surrounding the canonical binding site of mZO1-PDZ1 showed substantial CSP upon CalBioChem-322338. Thus, the unique molecular shape of NPL-4011 confined its binding to Dvl-PDZ in more specific manner.

### Assessment of Biological Activities of NPL-40XX Compounds

We assessed the inhibitory activity of the selected NPL-40XX compounds toward Wnt signaling pathways in the cultured-cell-based assay. For this purpose, we used BT-20 cell, a triple negative breast cancer (TNBC) cell line. Activation of Wnt signaling pathway is often observed in many cancers. Therefore, Wnt pathway inhibition is a potential therapeutic strategy (Polakis, 2012). Reportedly, activation of Wnt/β-catenin pathway has been observed in TNBC (Geyer et al., 2011; King et al., 2012a,b). For BT-20 cell, overexpression of Fzd 7 (Fzd7) has been reported; shRNA against Fzd7 suppresses the proliferation of BT-20 efficiently (Yang et al., 2011).

**FIGURE 8:**
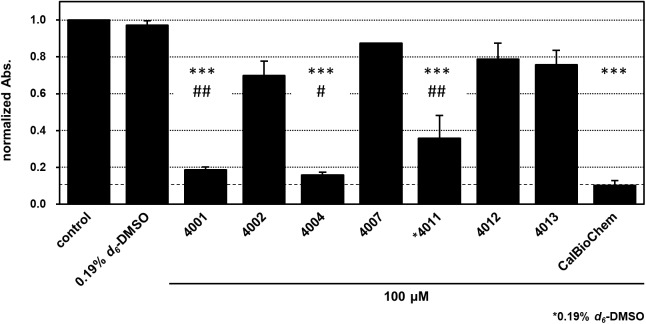
Cell proliferation inhibition of BT-20 triple negative breast cancer cell with indicated compounds, NPL-40XX and CalBioChem-322338. The cells were incubated with 100 μM indicated compounds including final 0.1% *d*_*6*_-DMSO. Control cells was incubated with the medium containing 0.1% *d*_*6*_-DMSO. NPL-4011 was examined in the presence of 0.19% *d*_*6*_-DMSO. The results of normalized absorbance of WST-8 assay with standard deviation were indicated. ^∗∗∗^*p* < 0.001 vs. comtrol (0.1 or 0.19% DMSO), ^##^*p* < 0.01 vs. CalBioCHem-322338, and ^#^*p* < 0.05 vs. CalBioCHem-322338, respectively.

We applied cell-based proliferation inhibition assay to concentrations of 100 μM of the compounds, including NPL-4001, 4002, 4004, 4007, 4011–4013, and the control compounds CalBioChem-322338 (**Figure 8** and **Supplementary Figure S5**). After 4 days of culture with 100 μM of the compounds, NPL-4001 and NPL-4004 showed approximately 80% inhibition of BT-20 cell proliferation, although NPL-4002, 4007, 4012, and 4013 showed no remarkable inhibitory activity. The stronger inhibitor NPL-4011 showed only 60% inhibition, which is less potent to 4001 and 4004. In this condition, the positive control CalBioChem-322338 showed better proliferation inhibitory activity, as 90% inhibition. The results demonstrated that our compound NPL-4011 must provide further improvement in terms of cell-based anticancer activity, although affinity against the target domain was highly optimized.

## Discussion

### Experimental Aspect of the NMR-Derived Docking Performance Index (NMR-DPI) for Dvl-PDZ Domain Inhibitor Screening

A common tradeoff that arises is that between accurate prediction of binding free energy Δ*G* in VS and the speed of calculation. Researchers must always confront the dilemmas of “rapidity–inaccuracy” and “sluggishness–accuracy” to process as many compounds as possible during a given period, simplified scoring functions should be chosen rather than the first principle-based force field in simulations between the target protein and ligands. In doing so, although such simplified scoring functions might all be equally inaccurate, eventually some scoring function can be expected to behave better than another for the specified library of the specified compounds. This study demonstrated an experimental strategy to select a better scoring function from the options presented by the GOLD program suite.

For this study, we used the averaged normalized chemical shift changes, Δδ_*ave*_, instead of *K_D_* for each of 17 training set molecules: 89% (15/17) of them bound to hDvl1-PDZ. According to theory, the maximum value of CSP should be recorded at the saturation point of titration by compounds. At that time, the maximum CSP might vary depending on the chemical structure of ligands. For example, aromatic rings in the ligand might induce larger CSPs upon binding because of the ring current effect. Another important shortcoming of CSP is that it is sensitive to the allosteric conformational changes of the target protein upon ligand binding. Consequently, generally, it is not recommended to use Δδ_*ave*_ (or other CSP-derived parameters) as an indication of *K_D_*. Irrespective of those shortcomings of CSP, however, we used Δδ_*ave*_ for this study based on the following two criteria. (1) Only compounds with similar chemical structure were analyzed and compared using Δδ_*ave*_. (2) Under the experimental conditions we used, the affinity of most ligands was weak. Moreover, they did not saturate to bind against hDvl1-PDZ at 1:2 molecular equivalence. We carefully assessed our experimental system using these two criteria. Finally, we inferred that if the criteria are satisfied, then the use of Δδ_*ave*_ as an indication of *K_D_* is convenient. Note that it was not feasible to use thermal shift assay to infer the affinity of the compounds in our case because many PDZ domains including hDvl1-PDZ showed no sharp *T_m_* transition curve. In addition, although the CSP experiment requires stable-isotope labeling, the experiment is less troublesome than those of the surface plasmon resonance experiment because it is unnecessary to immobilize the protein to the chip. Accordingly, information of amide NMR signals enables us to discern specific binding from non-specific binding.

### Comparison of Biological Activities of NPL-40XX Compounds

Results show that NPL-4011 has stronger affinity against hDvl1-PDZ *in vitro*, but it was a less potent cell growth inhibitor against BT-20 cell than CalBioChem-322338 was. To elucidate this observation in terms of bioavailability, we compared Lipinski’s drug-likeness parameters (Lipinski, 2000). The molecular weight of NPL-4011 (580.593) is greater than that of CalBioChem-322338 (373.388). The numbers of H-bond donors are equal (2), although the number of H-bond acceptors of NPL-4011 (8) is double that of CalBioChem-322338 (4). These two parameters violate Lipinski’s rule of five. Although the calculated log*P*-value (1.53 for NPL-4011 and 2.59 for CalBioChem-322338) is the only merit of NPL-4011, it did not contribute to overcoming the other shortcoming. Therefore, we infer that the poor biological activity of NPL-4011 is attributable to its bioavailability. This assumption is partially supported by our other observation. As described above, NPL-4001 and 4004 showed comparable growth inhibition activity to CalBioChem-322338. They are better than NPL-4011. Their Lipinski parameters are, respectively, 402.224 and 403.414 (MW), 1.75, and 2.05 (log*P*), 2 and 2 (H-bond donors), and 4 and 5 (H-bond acceptors). The numbers of donors and acceptors of H-bond are known to be crucially important to infer biological activity from the cell-based assay.

By contrast, NPL-4011 is expected to be more selective for Dvl-PDZ than the other PDZ domains in human cells because the crescent-shaped molecule fits to the unique cleft of Dvl-PDZ domains. The PDZ domain is the most abundant modular domain in human cell cytosol. Therefore, design of highly specific molecules to one specified PDZ domain might become crucially important. To satisfy both the specificity and the biological activity in terms of bioavailability, a good starting point is our new pharmacophore: bis-benzoic acid moiety. Screening smaller analogs such as NPL-4007 as the seed is better to improve the biological activity of this group of compounds. By contrast, a prodrug strategy starting from NPL-4011 is not recommended because it has already exceeded the drug-likeness parameters.

## Conclusion

In conclusion, we demonstrated a series of new class of compounds with higher affinity against hDvl1-PDZ. We proposed NMR-DPI as a useful experimental indication to optimize VS in the early stages of drug discovery.

## Author Contributions

KH and TT performed all the NMR titration experiments and discovered the inhibitors. KH also performed all the cell-based assays assisted by NG and AS. KA initiated the NMR signal assignment of hDvl1 and hDvl2 PDZ domains, whereas KH completed it. NG and TT prepared the plasmid constructs and protein samples of the optimized PDZ domains. The cell and developmental biologists MT and AS designed all the biological assays, set them up, and organized the biological part of the manuscript. HH constructed the focused library, developed the idea of NMR-based DPI, and performed VS. HH wrote the manuscript and organized the project.

## Conflict of Interest Statement

HH and TT are the founders of BeCellBar LLC., and TT is employed by BeCellBar LLC. The remaining authors declare that the research was conducted in the absence of any commercial or financial relationships that could be construed as a potential conflict of interest.

## References

[B1] Choi J. Ma S. Kim H.-Y. Yun J.-H. Heo J.-N. Lee W. (2016). Identification of small-molecule compounds targeting the dishevelled PDZ domain by virtual screening and binding studies. *Bioorg. Med. Chem.* 24 3259–3266. 10.1016/j.bmc.2016.03.026 27112452

[B2] Delaglio F. Grzesiek S. Vuister G. W. Zhu G. Pfeifer J. Bax A. (1995). NMRPipe: a multidimensional spectral processing system based on UNIX pipes. *J. Biomol. NMR* 6 277–293. 10.1007/BF00197809 8520220

[B3] Friesner R. A. Banks J. L. Murphy R. B. Halgren T. A. Klicic J. J. Mainz D. T. (2004). Glide: a new approach for rapid, accurate docking and scoring. 1. Method and assessment of docking accuracy. *J. Med. Chem.* 47 1739–1749. 10.1021/jm0306430 15027865

[B4] Fujii N. You L. Xu Z. Uematsu K. Shan J. He B. (2007). An antagonist of dishevelled protein-protein interaction suppresses -catenin-dependent tumor cell growth. *Cancer Res.* 67 573–579. 10.1158/0008-5472.CAN-06-2726 17234765

[B5] Fukunishi Y. Mikami Y. Nakamura H. (2005). Similarities among receptor pockets and among compounds: analysis and application to in silico ligand screening. *J. Mol. Graph. Model.* 24 34–45. 10.1016/j.jmgm.2005.04.004 15950507

[B6] Gao C. Chen Y. G. (2010). Dishevelled: the hub of Wnt signaling. *Cell. Signal.* 22 717–727. 10.1016/j.cellsig.2009.11.021 20006983

[B7] Geyer F. C. Lacroix-Triki M. Savage K. Arnedos M. Lambros M. B. MacKay A. (2011). B-Catenin pathway activation in breast cancer is associated with triple-negative phenotype but not with CTNNB1 mutation. *Mod. Pathol.* 24 209–231. 10.1038/modpathol.2010.205 21076461

[B8] Goda N. Tenno T. Takasu H. Hiroaki H. Shirakawa M. (2004). The PRESAT-vector: asymmetric T-vector for high-throughput screening of soluble protein domains for structural proteomics. *Protein Sci.* 13 652–658. 10.1110/ps.03439004 14978305 PMC2286733

[B9] Goddard T. D. Kneller D. G. (2004). *Sparky 3, 2004 University of California, San Francisco.* Available at: http://www.cgl.ucsf.edu/home/sparky/

[B10] Goodsell D. S. Morris G. M. Olson A. J. (1996). Automated docking of flexible ligands: applications of AutoDock. *J. Mol. Recognit.* 9 1–5. 10.1002/(SICI)1099-1352(199601)9:1<1::AID-JMR241>3.0.CO;2-6 8723313

[B11] Goto J. Kataoka R. Muta H. Hirayama N. (2008). ASEDock-docking based on alpha spheres and excluded volumes. *J. Chem. Inf. Model.* 48 583–590. 10.1021/ci700352q 18278891

[B12] Grandy D. Shan J. Zhang X. Rao S. Akunuru S. Li H. (2009). Discovery and characterization of a small molecule inhibitor of the PDZ domain of dishevelled. *J. Biol. Chem.* 284 16256–16263. 10.1074/jbc.M109.009647 19383605 PMC2713547

[B13] Hiroaki H. Umetsu Y. Nabeshima Y. Hoshi M. Kohda D. (2011). A simplified recipe for assigning amide NMR signals using combinatorial 14N amino acid inverse-labeling. *J. Struct. Funct. Genomics* 12 167–174. 10.1007/s10969-011-9116-0 21866395

[B14] Holland J. D. Klaus A. Garratt A. N. Birchmeier W. (2013). Wnt signaling in stem and cancer stem cells. *Curr. Opin. Cell Biol.* 25 254–264. 10.1016/j.ceb.2013.01.004 23347562

[B15] Huang Z. Wong C. F. (2016). Inexpensive method for selecting receptor structures for virtual screening. *J. Chem. Inf. Model.* 56 21–34. 10.1021/acs.jcim.5b00299 26651874

[B16] Ikura M. Kay L. E. Bax A. (1990). A novel approach for sequential assignment of 1H, 13C, and 15N spectra of larger proteins: heteronuclear triple-resonance three-dimensional NMR spectroscopy. Application to calmodulin. *Biochemistry* 29 4659–4667. 10.1021/bi00471a022 2372549

[B17] Jung Y.-S. S. Zweckstetter M. (2004). Mars - Robust automatic backbone assignment of proteins. *J. Biomol. NMR* 30 11–23. 10.1023/B:JNMR.0000042954.99056.ad 15452431

[B18] Kawabata T. Sugihara Y. Fukunishi Y. Nakamura H. (2013). LigandBox: a database for 3D structures of chemical compounds. *Biophysics* 9 113–121. 10.2142/biophysics.9.113 27493549 PMC4629684

[B19] King T. D. Suto M. J. Li Y. (2012a). The wnt/β-catenin signaling pathway: a potential therapeutic target in the treatment of triple negative breast cancer. *J. Cell. Biochem.* 113 13–18. 10.1002/jcb.23350 21898546 PMC10924801

[B20] King T. D. Zhang W. Suto M. J. Li Y. (2012b). Frizzled7 as an emerging target for cancer therapy. *Cell. Signal.* 24 846–851. 10.1016/j.cellsig.2011.12.009 22182510 PMC3268941

[B21] Kinoshita K. Murakami Y. Nakamura H. (2007). eF-seek: prediction of the functional sites of proteins by searching for similar electrostatic potential and molecular surface shape. *Nucleic Acids Res.* 35 W398–W402. 10.1093/nar/gkm351 17567616 PMC1933152

[B22] Lee H.-J. Wang N. X. Shao Y. Zheng J. J. (2009a). Identification of tripeptides recognized by the PDZ domain of Dishevelled. *Bioorg. Med. Chem.* 17 1701–1708. 10.1016/j.bmc.2008.12.060 19157887 PMC2713185

[B23] Lee H.-J. Wang N. X. Shi D.-L. Zheng J. J. (2009b). Sulindac Inhibits Canonical Wnt Signaling by Blocking the PDZ Domain of the Protein Dishevelled. *Angew. Chem. Int. Ed. Engl.* 48 6448–6452. 10.1002/anie.200902981 19637179 PMC2978498

[B24] Lindh M. Svensson F. Schaal W. Zhang J. Sköld C. Brandt P. (2015). Toward a benchmarking data set able to evaluate ligand- and structure-based virtual screening using public HTS data. *J. Chem. Inf. Model.* 55 343–353. 10.1021/ci5005465 25564966

[B25] Lipinski C. A. (2000). Drug-like properties and the causes of poor solubility and poor permeability. *J. Pharmacol. Toxicol. Methods* 44 235–249. 10.1016/S1056-8719(00)00107-6 11274893

[B26] McGann M. (2011). FRED pose prediction and virtual screening accuracy. *J. Chem. Inf. Model.* 51 578–596. 10.1021/ci100436p 21323318

[B27] McGaughey G. B. Sheridan R. P. Bayly C. I. Culberson J. C. Kreatsoulas C. Lindsley S. (2007). Comparison of topological, shape, and docking methods in virtual screening. *J. Chem. Inf. Model.* 47 1504–1519. 10.1021/ci700052x 17591764

[B28] Motono C. Nakata J. Koike R. Shimizu K. Shirota M. Amemiya T. (2011). SAHG, a comprehensive database of predicted structures of all human proteins. *Nucleic Acids Res.* 39 D487–D493. 10.1093/nar/gkq1057 21051360 PMC3013665

[B29] Polakis P. (2012). Drugging Wnt signalling in cancer. *EMBO J.* 31 2737–2746. 10.1038/emboj.2012.126 22617421 PMC3380214

[B30] Reya T. Clevers H. (2005). Wnt signalling in stem cells and cancer. *Nature* 434 843–850. 10.1038/nature03319 15829953

[B31] Schrödinger L. L. C. (2015). *The {PyMOL} Molecular Graphics System, Version∼1.8.*

[B32] Shan J. Shi D.-L. Wang J. Zheng J. (2005). Identification of a Specific Inhibitor of the Dishevelled PDZ Domain †. *Biochemistry* 44 15495–15503. 10.1021/bi0512602 16300398

[B33] Takebe N. Harris P. J. Warren R. Q. Ivy S. P. (2011). Targeting cancer stem cells by inhibiting Wnt, Notch, and Hedgehog pathways. *Nat. Rev. Clin. Oncol.* 8 97–106. 10.1038/nrclinonc.2010.196 21151206

[B34] Tannishtha R. Morrison S. J. Clarke M. F. Weissman I. L. (2001). Stem cells, cancer, and cancer stem cells. *Nature* 414 105–111. 10.1007/978-1-60327-933-8 11689955

[B35] Tenno T. Goda N. Umetsu Y. Ota M. Kinoshita K. Hiroaki H. (2013). Accidental interaction between PDZ domains and diclofenac revealed by NMR-assisted virtual screening. *Molecules* 18 9567–9581. 10.3390/molecules18089567 23966078 PMC6270271

[B36] Trott O. Olson A. J. (2010). AutoDock Vina. *J. Comput. Chem.* 31 445–461. 10.1002/jcc.21334 19499576 PMC3041641

[B37] Umetsu Y. Goda N. Taniguchi R. Satomura K. Ikegami T. Furuse M. (2007). Assignment of 1H, 13C and 15N resonances of N-terminal domain of DnaA protein. *Biomol. NMR Assign.* 1 57–59. 10.1007/s12104-007-9015-2 19636826

[B38] Umetsu Y. Goda N. Taniguchi R. Satomura K. Ikegami T. Furuse M. (2011). 1H, 13C, and 15N resonance assignment of the first PDZ domain of mouse ZO-1. *Biomol. NMR Assign.* 5 207–210. 10.1007/s12104-011-9301-x 21431884

[B39] Verdonk M. L. Cole J. C. Hartshorn M. J. Murray C. W. Taylor R. D. (2003). Improved protein-ligand docking using GOLD. *Proteins* 52 609–623. 10.1002/prot.10465 12910460

[B40] Visvader J. E. Lindeman G. J. (2008). Cancer stem cells in solid tumours: accumulating evidence and unresolved questions. *Nat. Rev. Cancer* 8 755–768. 10.1038/nrc2499 18784658

[B41] Wang Z. Sun H. Yao X. Li D. Xu L. Li Y. (2016). Comprehensive evaluation of ten docking programs on a diverse set of protein-ligand complexes: the prediction accuracy of sampling power and scoring power. *Phys. Chem. Chem. Phys.* 18 12964–12975. 10.1039/c6cp01555g 27108770

[B42] Williamson M. P. (2013). Using chemical shift perturbation to characterise ligand binding. *Prog. Nucl. Magn. Reson. Spectrosc.* 73 1–16. 10.1016/j.pnmrs.2013.02.001 23962882

[B43] Wong H.-C. Bourdelas A. Krauss A. Lee H.-J. Shao Y. Wu D. (2003). Direct binding of the PDZ domain of Dishevelled to a conserved internal sequence in the C-terminal region of Frizzled. *Mol. Cell* 12 1251–1260. 10.1016/S1097-2765(03)00427-1 14636582 PMC4381837

[B44] Yang L. Wu X. Wang Y. Zhang K. Wu J. Yuan Y.-C. (2011). FZD7 has a critical role in cell proliferation in triple negative breast cancer. *Oncogene* 30 4437–4446. 10.1038/onc.2011.145 21532620

[B45] Zhang Y. Appleton B. A. Wiesmann C. Lau T. Costa M. Hannoush R. N. (2009). Inhibition of Wnt signaling by Dishevelled PDZ peptides. *Nat. Chem. Biol.* 5 217–219. 10.1038/nchembio.152 19252499

