# Supplementary material for: Discovery of Potent Disheveled/Dvl Inhibitors Using Virtual Screening Optimized With NMR-Based Docking Performance Index
Source: Front Pharmacol. 2018 Sep 5;9:983. doi: 10.3389/fphar.2018.00983 (PMC6134994; doi:10.3389/fphar.2018.00983)
Supplement: Supplementary file 1 [file Data_Sheet_1.pdf]

Supplementary Materials

Discovery of potent Dishevelled/Dvl inhibitors using virtual screening optimized with NMR-based docking performance index

Kiminori Hori[1†], Kasumi Ajioka[2], Natsuko Goda[1], Asako Shindo[3], Maki Takagishi[4], Takeshi Tenno[1,5], and Hidekazu Hiroaki[1,2,5,*]

[1] Laboratory of Structural Molecular Pharmacology, Graduate School of Pharmaceutical Sciences, Nagoya University, Nagoya 464-8601, Aichi, Japan

[2] Department of Biological Science, School of Science, Nagoya University, Nagoya 464-8601, Aichi, Japan

[3] Division of Biological Science, Graduate School of Science, Nagoya University, Nagoya 464-8601, Aichi, Japan

[4] Department of Pathology, Graduate School of Medicine, Nagoya University, Nagoya, 466-8550, Aichi, Japan

[5] BeCellBar LLC, Business Incubation Center, Nagoya University, Aichi, Nagoya 464-8601, Aichi, Japan

*** Correspondence:**
Hidekazu Hiroaki, hiroaki.hidekazu@f.mbox.nagoya-u.ac.jp

Supplementary Material

Supplementary Table S1

Supplementary Figure S1

Supplementary Figure S2

Supplementary Figure S3

Supplementary Figure S4

Supplementary Figure S5

Supplementary Table S1

Compound ID (NPL-)	$\Delta\delta_{ave.}$ / ppm	GOLD Score	Compound ID (NPL-)	$\Delta\delta_{ave.}$ / ppm	GOLD Score
**4001**	0.089	49.23*	**4011**	0.058	98.59
**4002**	0.073	51.53*	**4012**	0.043	95.83
4003	0.003	60.09	**4013**	0.037	98.47
**4004**	0.030	60.84	**4014**	0.012	86.14
4005	0.002	46.60	**4015**	0.017	83.81
4006	0.003	59.76	4016	0.005	59.52
**4007**	0.025	64.39			
4008[1]	0.018		[1] CalBioChem-322338		
4009[2]	0.003	-	[2] sulindac		
4010[3]	0.003	-	[3] BMD4702		

Normalized total CSPs of hDvl1-PDZ induced by 2.0 eq. of the advanced Dvl1-PDZ binding compounds. GOLD scores are also indicated as the score with consensus scoring of the function GS-CS. The compounds with $\Delta\delta_{ave.}$ larger than 0.010 are marked as bold, which were judged as potential binding. Although NPL-4001 and 4002 showed lower docking scores than the other, the compounds were assessed because they possessed Br atom in the amino-benzoic acid moiety. NPL-4008 ~ 4010 are the reported Dvl binders, CalBioChem-322338, sulindac, and BMD4702, respectively.

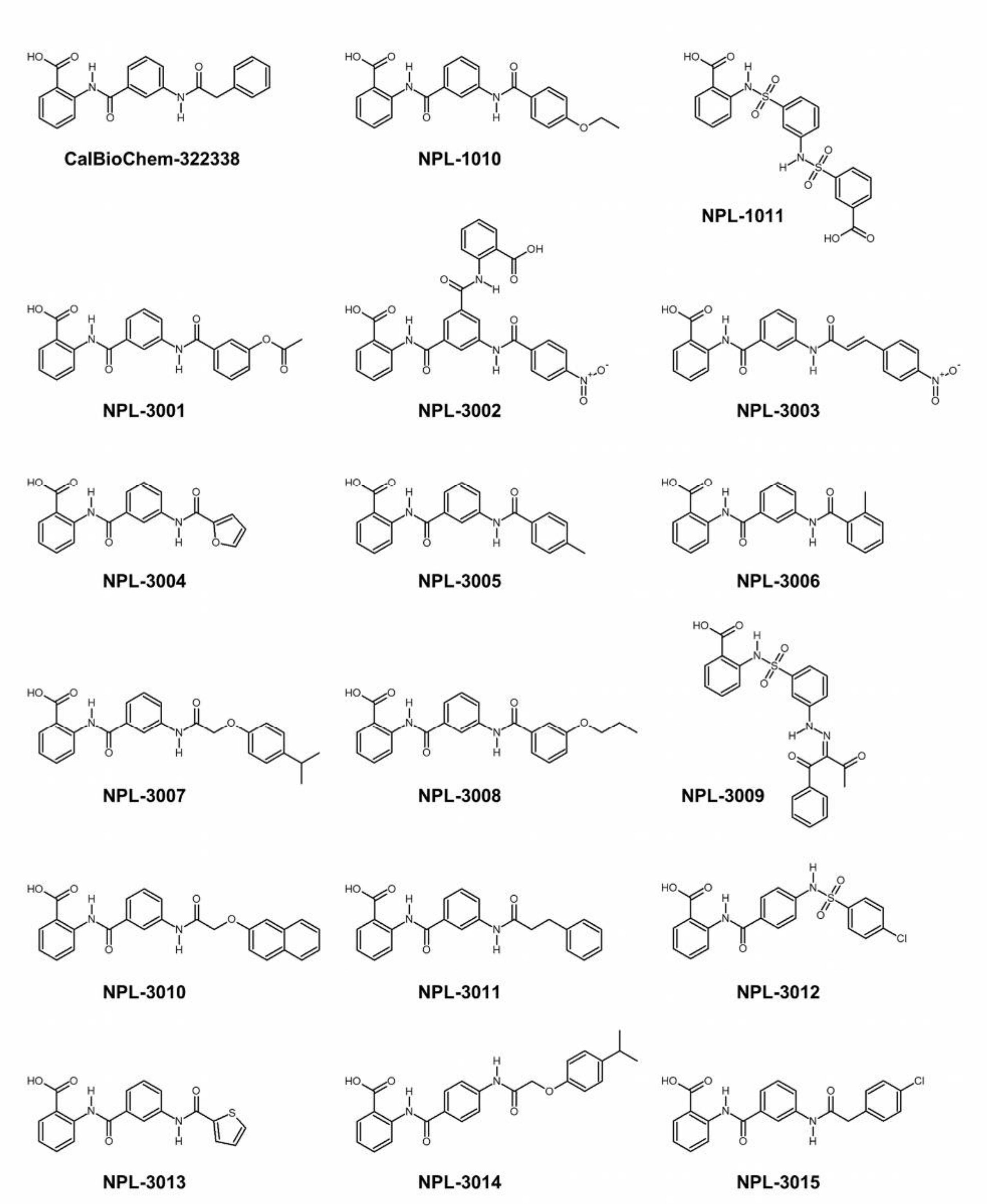

Supplementary Figure S1

Full list of the prototypical PDZ domain-binding compounds used in the study with CalBioChem-322338.

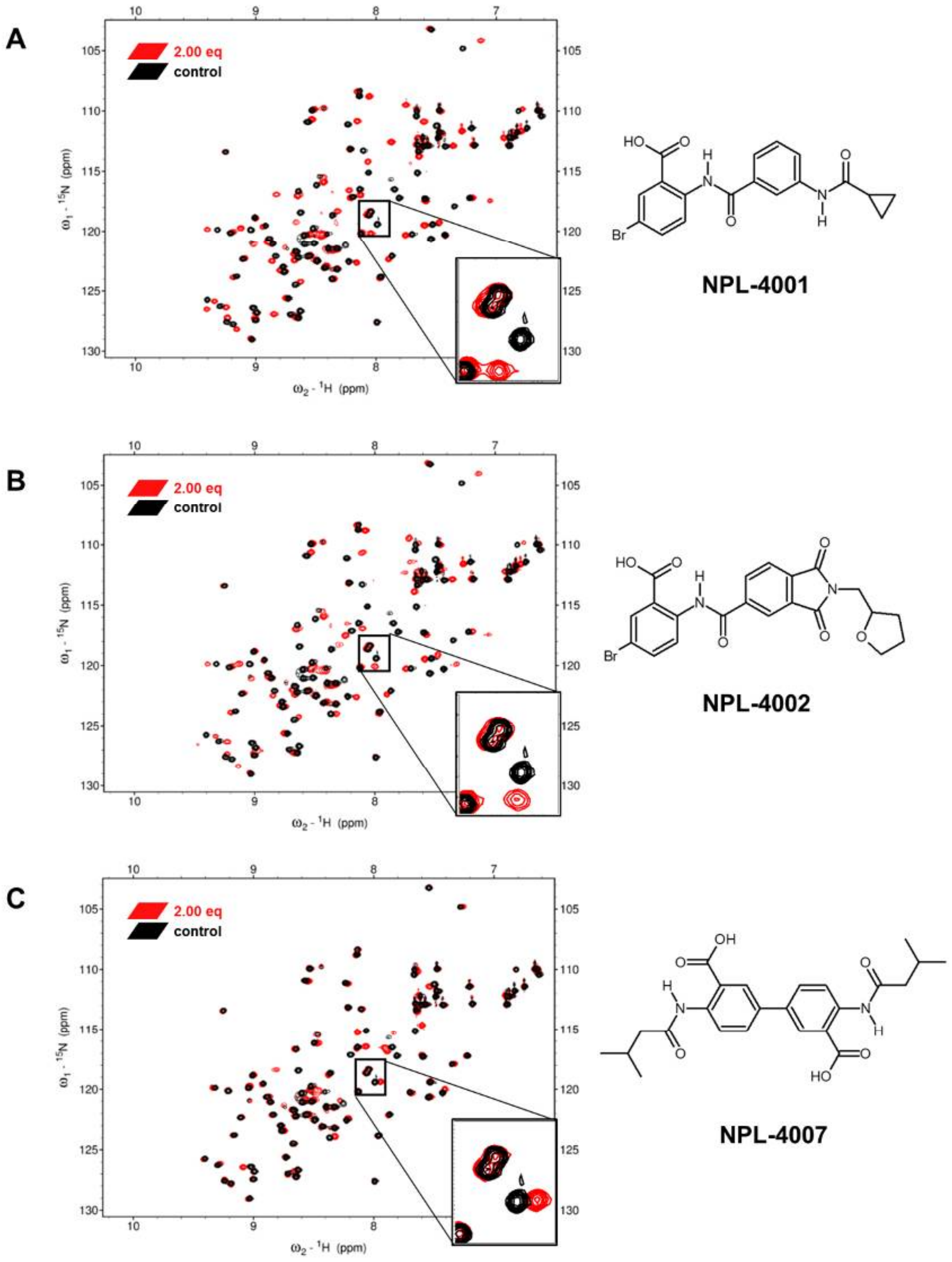

Supplementary Figure S2

Expanded region of ${}^{1}H$-${}^{15}N$ HSQC spectra of hDvl1-PDZ for the NMR titration experiment of hDvl1-PDZ with NPL-4001 (A), 4002 (B), and 4007 (C), with the corresponding chemical structures. The spectra with 0 eq. (black) and 2.0 eq. (red) of the indicated ligand were overlaid.

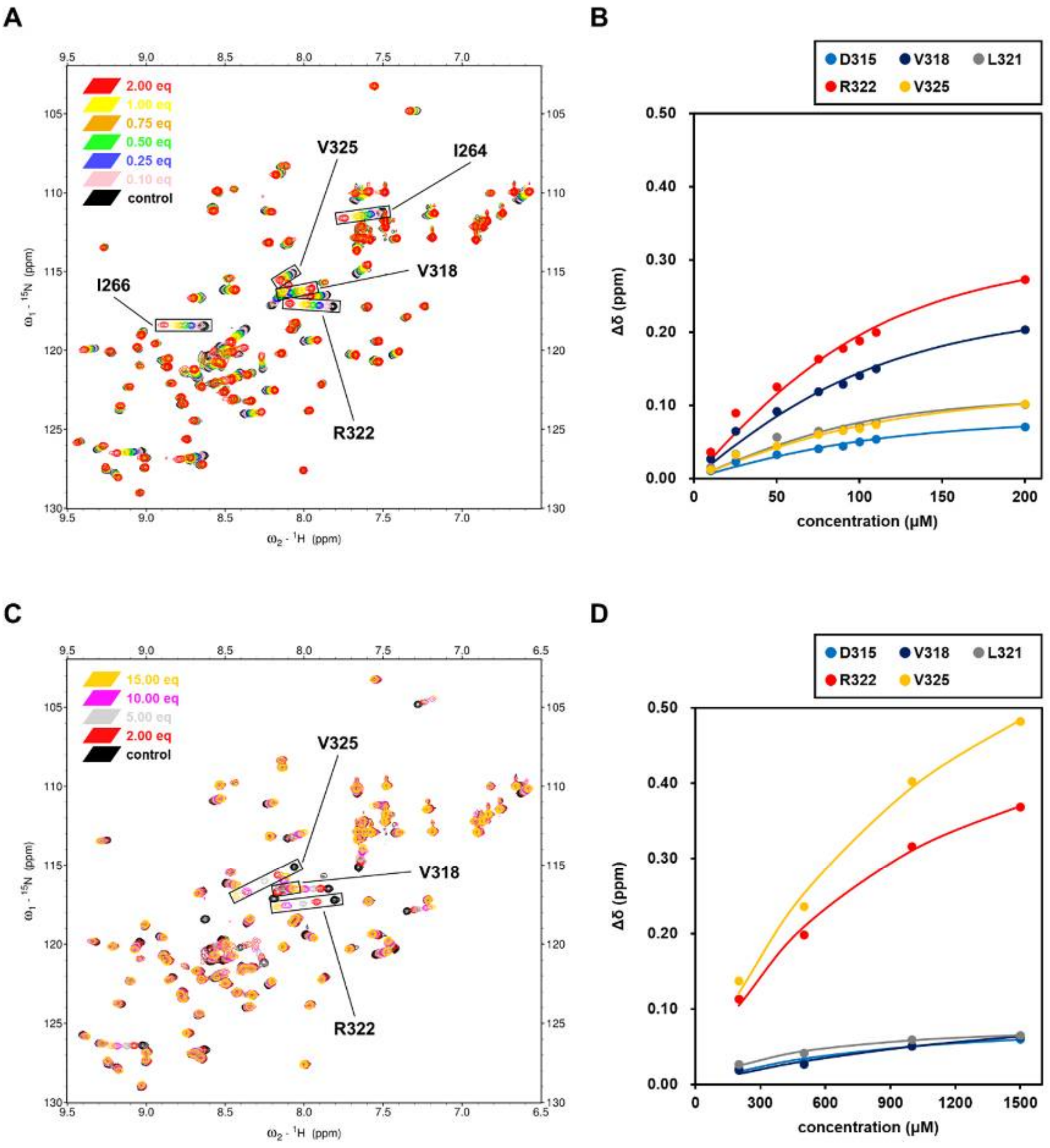

Supplementary Figure S3

Comparison of the NMR titration experiment of hDvl1-PDZ with NPL-4011 (A, B) and CalBioChem-322338 (C,D). The assignments of the signal series are labeled. A. Expanded region of ^{1}H-^{15}N HSQC spectra of hDvl1-PDZ with 0 eq. (black), 0.25 eq. (pink), 0.5 eq. (navy), 0.75 eq. (green), 1.0 eq. (orange), 1.2 eq. (yellow) and 2.0 eq. (red) of NPL-4011 were overlaid. B. Expanded region of ^{1}H-^{15}N HSQC spectra of hDvl1-PDZ with 0 eq. (black), 2.0 eq. (red), 5.0 eq. (grey), 10 eq. (magenta) and 15 eq. (orange) of CalBioChem-322338 were overlaid. C. Normalized chemical shift changes of the selected hDvl1-PDZ residues upon titration with NPL-4011 were plotted against the ligand concentration. Solid lines indicate the non-linear fitting curves of each signals based on the single-site binding model. D. Normalized chemical shift changes of the selected hDvl1-PDZ residues upon titration with CalBioChem-322338 were plotted against the ligand concentration.

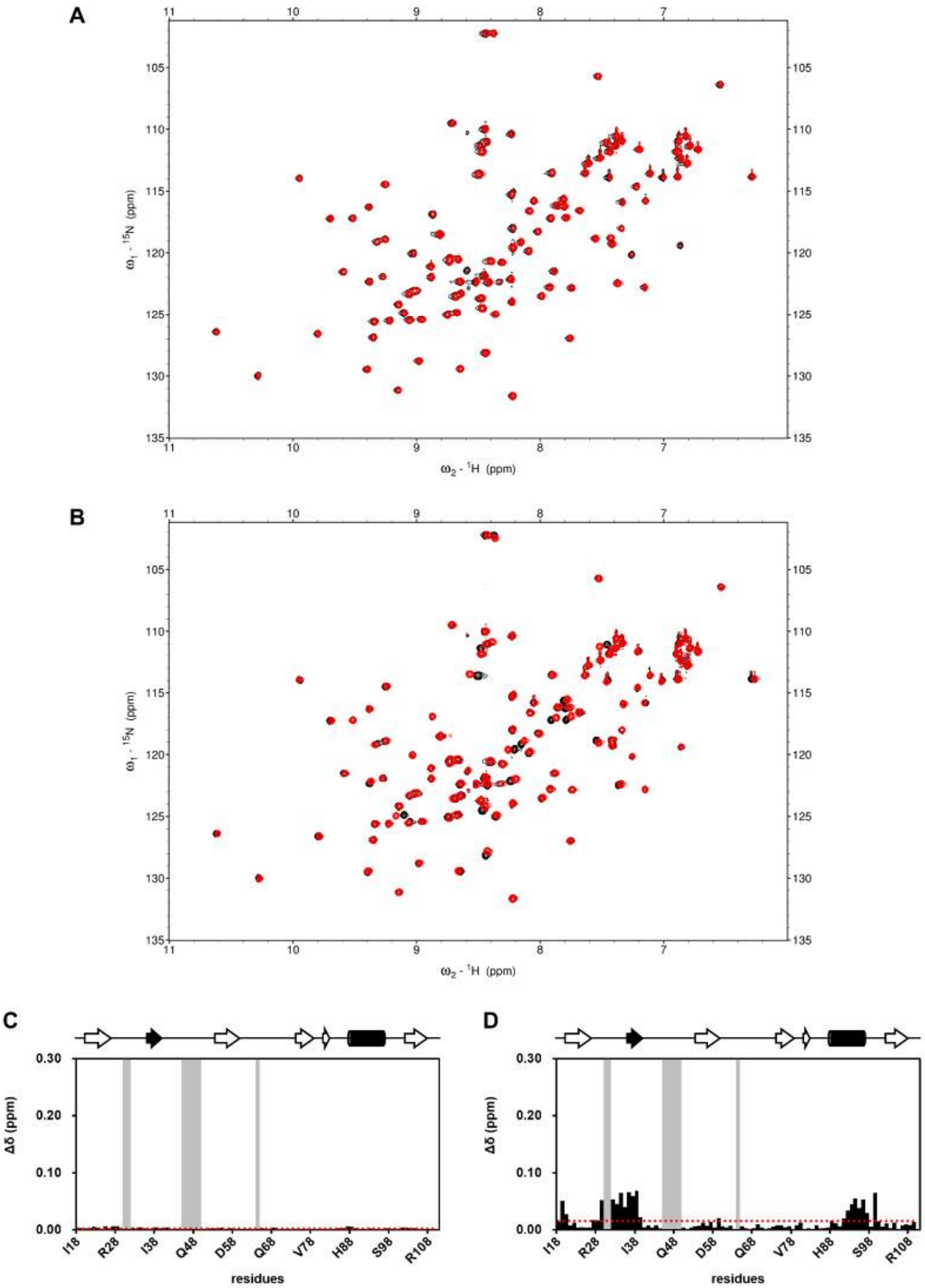

Supplementary Figure S4

Comparison of the NMR titration experiment of mZO1-PDZ1 with NPL-4011 (A, C) and CalBioChem-322338 (B,D). The assignments of the signal series are labeled. A. ^{1}H-^{15}N HSQC spectra of mZO1-PDZ1 with 0 eq. (black), and 2 eq. (red) of NPL-4011 were overlaid. C. ^{1}H-^{15}N HSQC spectra of mZO1-PDZ1 with 0 eq. (black), and 2 eq. (red) of CalBioChem-322338 were overlaid. B, D; normalized chemical shift changes Δδ is plotted against residue numbers. Gray residues are missing or unassigned residues, and white residues indicate Pro. The secondary structure of mZO1-PDZ1 is shown at the top of the figures, whereas α2 and β2 are shown in black.

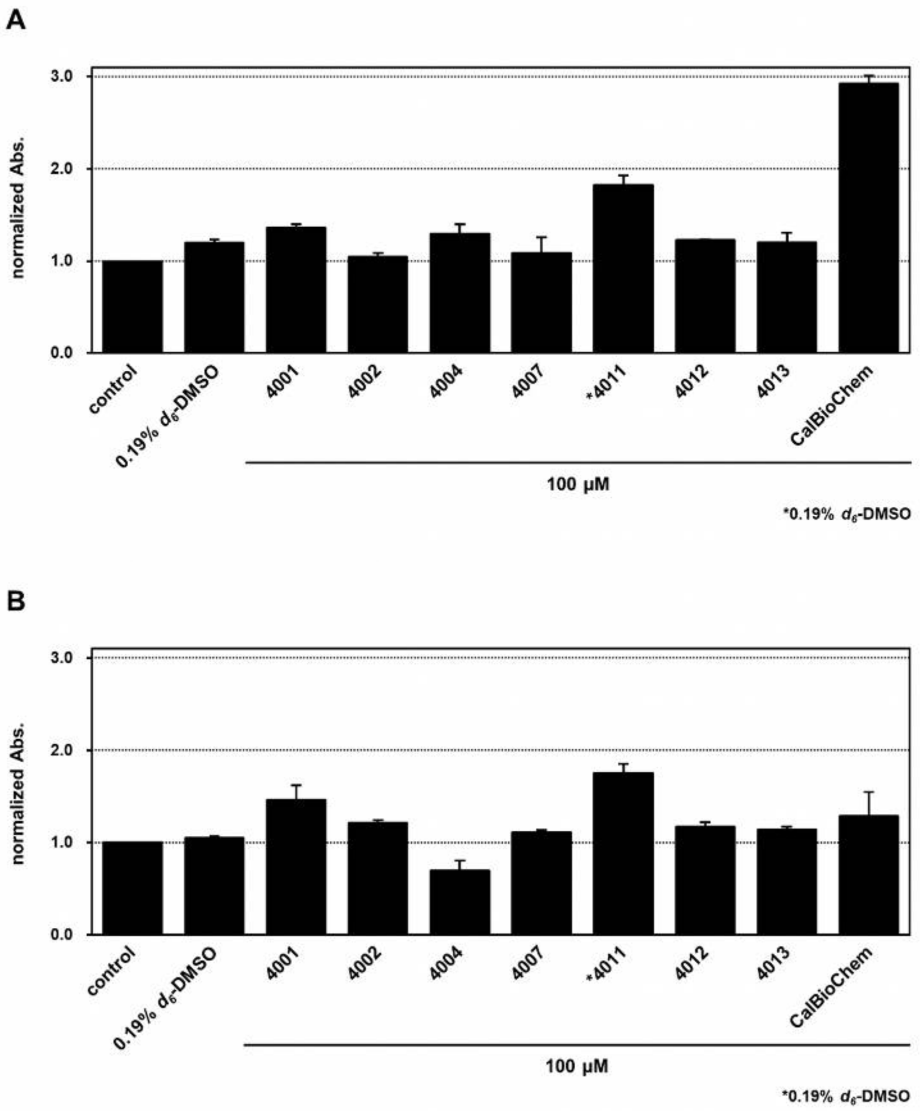

Supplementary Figure S5

Cytotoxicity of the compounds, NPL-40XX and CalBioChem-322338 against BT-20 triple negative breast cancer. Cells were treated with 100 μM of the compounds for 48 h (A) and 96 h (B) including final 0.1% d_6-DMSO. LDH activity assay was performed to evaluate comparative cytotoxicity. BT-20 triple negative breast cancer cell with indicated compounds, NPL-40XX and CalBioChem-322338. Control cells was incubated with the medium containing 0.1% d_6-DMSO. NPL-4011 was examined in the presence of 0.19% d_6-DMSO. The results of normalized absorbance at 490 nm of LDH activity assay with standard deviation were indicated.
